# *CRM1* expression: association with high prognostic value in laryngeal cancer

**DOI:** 10.55730/1300-0144.5655

**Published:** 2023-02-03

**Authors:** Talih ÖZDAŞ, Sibel ÖZDAŞ, İpek CANATAR, Erdal ÇOŞKUN, Elif Burcu ŞENYURT, Orhan GÖRGÜLÜ

**Affiliations:** 1 Department of Otorhinolaryngology, Adana City Training and Research Hospital, Health Science University, Adana, Turkiye; 2 Department of Bioengineering, Faculty of Engineering Sciences, Adana Alparslan Türkeş Science and Technology University, Adana, Turkiye; 3 Genomics Team, Microsoft Research, Redmond, WA, USA

**Keywords:** Chromosome region maintenance 1, laryngeal cancer, clinicopathological significance, prognosis

## Abstract

**Background/aim:**

Laryngeal cancer is a very common malignant tumor of the head and neck. While laryngeal cancer does not show any obvious early symptoms, it tends to have a poor prognosis in advanced clinical stages. Chromosome region maintenance 1 (CRM1) mediates the nuclear export of some RNAs, major and tumor suppressor proteins and has been associated with the pathogenesis of many tumors. However, the clinicopathological significance of *CRM1* gene expression in laryngeal cancer has not been clarified yet. Therefore, this study aims to detect the expression of *CRM1* in laryngeal cancer and to investigate its relationship with clinicopathological parameters and prognosis.

**Materials and methods:**

*CRM1* expression in matched tumor and normal tissues obtained from 43 laryngeal cancer patients were evaluated intracellular for protein and mRNA levels by immunohistochemical staining (IHC), western-blot, and quantitative real–time RT-PCR (qRT-PCR), respectively.

**Results:**

IHC, western-blot, and qRT-PCR analyses showed that CRM1 expression was significantly increased in laryngeal cancer tissue compared to normal tissue. Increased expression of CRM1 has been associated with poor prognostic clinicopathological features, including advanced tumor stage, increased tumor invasion, larger tumor size, positive lymph node metastasis, distant metastasis, and invasive histological type by IHC, western-blot, and qRT-PCR. Kaplan–Meier survival analysis showed that patients with high expression of *CRM1* exhibited lower overall survival compared to those with low expression (Log-rank = 7.16, p = 0.007). According to the The Cancer Genome Atlas (TCGA) datasets, elevated *CRM1* expression in head and neck cancer including cases of squamous cell laryngeal origin is associated with advanced tumor stage and histological grade (p > 0.05, for all).

**Conclusion:**

Consequently, *CRM1* plays an important role in laryngeal cancer and may serve as an indicator and prognostic factor for poor overall survival in laryngeal cancer patients.

## 1. Introduction

Laryngeal cancer is the second most common malignant tumor of head and neck cancer (HNC) with a high incidence rate [1]. According to histological typing, more than 90% of laryngeal cancers are malignant squamous cell carcinoma lesions [2]. Laryngeal cancer is aggressive and invasive, with a 5-year survival rate of more than 80% in the early stages. However, this rate is 30%–40% in the presence of lymph node metastases, and less than 20% in distant metastases [3,4]. In laryngeal cancer, tumor size, primary tumor invasion, lymph node involvement, distant metastases, and histological grading are clinically used prognostic factors to determine the behavior of the disease, the mortality rate, or success level of treatment [5,6]. Recurrence and metastasis are still a big challenge for patients with laryngeal cancer, and the long-term survival rate is still low [5–7]. Therefore, there is a need to investigate new molecular markers that can identify patients with poor prognosis and used in the development of targeted therapies for advanced laryngeal cancer.

Chromosome region maintenance 1 (CRM1; EXPORTIN 1, XPO1) is a member of karyopherins that mediates the nuclear export of some classes of RNA, of 200 mammalian cargo proteins including tumor suppressor proteins and oncogenic proteins in a ras-related nuclear protein guanosine triphosphatase (Ran/GTP)-dependent manner [8–10]. CRM1 is also involved in centrosome proliferation and spindle assembly, maintenance of chromosome structure, and cell cycle regulation [11,12]. CRM1 was reported to be overexpressed in many human cancers and its overexpression is associated with aggressive behavior and poor survival [13–18]. The biological role of CRM1 in laryngeal cancer have not yet been evaluated [8–10,19].

In this study, the expression of *CRM1* at the intracellular, protein, and mRNA levels and its relationship with clinicopathologic parameters in laryngeal cancer were evaluated to further elucidate the relationship between *CRM1* and the clinical features of laryngeal cancer and to investigate its clinical prognostic value.

## 2. Materials and methods

### 2.1. Patient specimen collection

The study included 43 patients with suspicion of laryngeal cancer in clinical and radiological examination, and histopathologically confirmed diagnosis of squamous cell laryngeal cancer with biopsy. There were 43 admitted patients of laryngeal cancer from January 2017 to February 2022 in Department of ENT, The Adana City Training and Research Hospital, Health Science University. This prospective study was approved by the local ethical review board (No: 406). All fresh tumor and normal tissue samples were collected, with the patient’s written and verbal consent, at the time of surgery. The 43 adjacent nontumor tissues matched with the same patients with laryngeal cancer were chosen as controls. Normal tissue samples were obtained at least 5 mm apart from tumor margin [20,21]. Tumor/normal differentiation of tissues was performed with macroscopic and microscopic examinations by the pathologists and then used in the study. The staging system recommended by the World Health Organization (WHO) standard and the American Joint Committee on Cancer (AJCC) was used for clinical data [5,6]. The primary tumor size was calculated based on Murphy et al.’s method by measuring the three orthogonal diameters obtained from specimen after surgical resection [22]. Overall survival (OS) was defined as the time from curative surgery to his/her death date or final clinical follow-up to (average survival time: 55.4 months; range: 30–60 months). All patients were followed up with polyclinic visits and phone calls after the surgery. Patients who were treated with chemotherapy, immunotherapy and/or radiotherapy, are serology-positive, have second primary neoplasia, and history of another malignancy, and have nonsquamous cell laryngeal cancer were excluded from the study.

### 2.2. Hematoxylin and Eosin (H&E) staining

Hematoxylin and Eosin (H&E) was studied in two groups as cancer tissue and adjacent normal tissue specimens taken from patients. Larynx tissue samples were fixed with 10% formaldehyde for 24 h, passed through ethanol series for fixation, transparentized with xylene and embedded in paraffin blocks. Sections of 5 μm thickness were taken from paraffin blocks. Sections were stained with H&E (ab245880, Abcam, Cambridge, UK) and examined histopathologically under a microscope (Aperio CS2, Leica, Germany) with a slide at 200× magnification [23].

### 2.3. Immunohistochemical staining (IHC)

Immunohistochemical staining (IHC) was performed to determine the intracellular expression of the CRM1 protein in laryngeal tissue samples [24]. The specimens were taken from paraffin-embedded tissue blocks. For the deparaffinization, the slides were left in xylol and then passed through a series of alcohol. Dehydrated tissues were washed with phosphate-buffered saline (PBS) twice and exposed to 3% H_2_O_2_ for 15 min. The nonspecific binding was prevented by the application of UltraV block (TP-060-HL; NeoMarker, Fremont, CA). The slides were incubated with CRM1 (1:100; ab191081, Abcam, Cambridge, MA, USA) as the first antibody overnight at 4 °C [15,25]. They were then incubated for 45 min at 37 °C with Goat Anti-Rabbit IgG H&L (HRP) (1:1000; ab205718, Abcam, Cambridge, MA, USA) as the secondary antibody. The slides were exposed to the streptavidin peroxidase enzyme complex for 20 min, and 3-amino-9-ethylcarbazole was added as a chromogen and incubated for 10 min. Each slide was examined with a microscope at 200× magnification. Intensity of uptake was semiquantitatively scored as 0 (0, no involvement), 1 (+, weak immunoreactivity), 2 (++, moderate immunoreactivity), 3 (+++, strong immunoreactivity). The percentage of uptake was scored as 1 (0%–10%, focal), 2 (11%–50%, regional), and 3 (51%–100%, diffuse), as the ratio of cells/structures with immunoreactivity to the total cells/structures. The IHC staining scoring was calculated following the formula below:


HistoSCORE=ΣPi (i+1)   (i: degree of staining, Pi: percentage of uptake).

### 2.4. Western-blot

Western-blot analysis was performed to determine the expression of the CRM1 protein in laryngeal tissue samples. Tissue samples were homogenized with the TissueLyser LT (Qiagen, Hilden, Germany). Proteins were isolated using radioimmunoprecipitation lysis buffer (R0278, Sigma, USA) and protease inhibitor cocktail (P8340, Sigma, USA). Quick Start Bradford Protein Assay Kit (Bio-Rad, Germany) was used to determine total protein concentration. Proteins with lysis buffer were loaded onto 10% sodium dodecyl-sulfate polyacrylamide gel electrophoresis (SDS-PAGE) and transferred to polyvinylidene difluoride) membrane (PVDF). The membranes were incubated with primary antibodies CRM1 (1:1000; ab191081; Abcam, USA) and Beta Actin (ACTB) (1:1000; ab8226, Abcam, USA) at 4 °C overnight. CRM1 and ACTB primary antibodies were incubated at room temperature using Goat Anti-Rabbit IgG HRP and Goat Anti-Mouse IgG HRP (1:2000; ab205719; Abcam, Cambridge, MA, USA) in 1X tris buffer saline with Tween 20 (TBST). The specific CRM1 protein became visible through the chemiluminescent agent (ECL, Bio-Rad, Hercules, USA) and the amount of protein was detected [15]. The relative density of the protein bands was analyzed densitometrically with ImageLab software (Bio-Rad, Hercules, USA) [25–27].

### 2.5. Quantitative real–time RT-PCR (qRT-PCR)

The quantitative real–time RT-PCR (qRT-PCR) method was utilized to determine the expression of the *CRM1* gene at the relative mRNA level in laryngeal tissue samples. Trizol (93289, SigmaAldrich, Germany) was used for the isolation of total RNA. Complementary DNA (cDNA) synthesis was performed with the RevertAid First Strand cDNA kit (K1622, Thermo Scientific, USA) using isolated RNA. NormFinder (https://www.moma.dk/normfinder-software) was performed to determine the most suitable reference gene and/or gene combinations among five reference genes *ACTB*, *5*′*-Aminolevulinate Synthase 1 (ALAS1)*, *Glucuronidase Beta (GUSB)*, *Hydroxymethylbilane Synthase (HMBS)*, *Ribosomal Protein L29 (RPL29)]* for *CRM1* gene normalization in cancer and healthy tissue samples [28–31]. The primer sets used in the amplification respectively forward and reverse: *CRM1* 5′-gggaaaactgaaacccacct-3′ and 5′-ctgaaatcaagcagctgacg-3′ (242 bp); *ACTB* 5′-agaaaatctggcaccacacc-3′ and 5′-tagcacagcctggatagcaa-3′ (173 bp); *ALAS1* 5′-ggcagcacagatgaatcaga-3′ and 5′-cctccatcggttttcacact-3′ (150 bp); *GUSB* 5′-agccagttcctcatcaatgg-3′ and 5′-ggtagtggctggtacggaaa-3′ (160 bp); *HMBS* 5′-agtgtggtgggaaccagc-3′ and 5′-caggatgatggcactgaactc-3′ (144 bp); *RPL29* 5′-ggcgttgttgaccctatttc-3′ and 5′-gtgtgtggtgtggttcttgg-3′ (120 bp). For qRT-PCR, Rotor-Geneq (Qiagen, Germany) device was used and the manufacturer’s protocol was followed while using the SYBR Green PCR kit (Roche Applied Science, Germany) [25,32]. Baseline and cycle thresholds (Ct values) for each gene were determined using Rotor Gene Q Software 1.2 (Qiagen, Germany). The relative value of *CRM1* mRNA expression level was calculated using the 2-^ΔΔCt^ method [2].

### 2.6. The Cancer Genome Atlas (TCGA) data analysis

Laryngeal cancer is the second most common malignant tumor of the HNC [1]. Accordingly, *CRM1* mRNA upregulation in HNC was analyzed by cBioPortal [33]. It was utilized to analyze the relationship between *CRM1* mRNA expression and clinicopathological parameters (Neoplasm AJCC Clinical Group Stage) in HNC downloaded from The Cancer Genome Atlas (TCGA).

### 2.7. Statistical analysis

SPSS v24.0 (Inc., Chicago, IL, USA) and Prism 8 (GraphPad Software, Inc., La Jolla, CA, USA) software packages were used for all statistical analyses. The NormFinder (https://www.moma.dk/normfinder-software) was performed to determine the most suitable reference gene and/or its combinations for *CRM1* gene best normalization in the qRT-PCR method [30,31]. Two groups were established according to the cut-off value in analysis of the associations between patient clinic parameters and gene expression level. The best cut-off value for *CRM1* expression at the mRNA and protein level was determined using X-tile software (best p-value) [34]. The correlations between *CRM1* expression level and clinicopathological variables in laryngeal cancer were analyzed using Pearson’s chi-squared and Fisher’s exact test. The Mann–Whitney U or Student’s test was used for comparing of two groups. For more than two groups, comparison of numerical variables was performed using the one-way ANOVA or Kruskal–Wallis test. Bonferroni correction was used for the post hoc tests. Kaplan–Meier and log-rank (Mantel Cox) tests were used for overall survival analysis. All experiments were performed in triplicate. Categorical variables were expressed as numbers and percentages, and numerical variables were expressed as mean ± standard deviation (SD). p < 0.05 represents significance.

## 3. Results

### 3.1. Clinicopathological characteristics of laryngeal cancer patients

The demographic and clinicopathological characteristics of the patients included in the study are shown in Table 1. The age range of seventeen of the patients (39.5%) was 31–59 (mean age 51.31 ± 4.89). The age range of twenty-six of the patients (60.5%) was 60–71 (mean age 64.31 ± 3.40). Considering all patients without age grouping, the mean age was 59.36 ± 7.52. The tumor size was 5 cm^3^ or less in 24 of the patients (55.8%) (size range 0.9–5 cm^3^, mean size of 2.87 ± 1.26 cm^3^) and above 5 cm^3^ in 19 of the patients (44.2%) (size range 5–12.5 cm^3^, mean size was 7.97 ± 1.83 cm^3^). The mean tumor size of all patients was 5.13 ± 2.97 cm^3^.

### 3.2. Histopathology of laryngeal cancer and normal tissues by H&E staining

**Figure 1:**
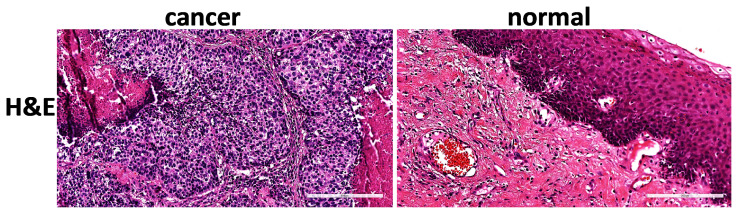
H&E staining data of matched tumor and normal tissue sections of laryngeal cancer patients. Compared to normal tissues, increased necrosis, increased cell nucleus cytoplasm ratio, chromatin condensation, lymphocyte and plasma cell-rich inflammation in desmoplastic fibrous stroma were observed in laryngeal tumor tissues (left 100× magnification, scale bar = 300 μm and right 200× magnification, scale bar = 200 μm). H&E: Hematoxylin-Eosin.

Laryngeal tumor and matched normal tissues were stained with H&E to evaluate pathological morphology and cell growth characteristics (Figure 1). Loss of polarity, dyskeratosis, keratin pearls, intercellular bridges, increased cell nucleus-to-cytoplasm ratio, increased nuclear chromatin, prominent eosinophilic nucleoli, and atypical mitosis were observed in tumor tissues compared to normal tissues. There is an increase in necrosis and inflammation richness in lymphocytes and plasma cells in the desmoplastic fibrous stroma.

### 3.3. Intracellular expression of CRM1 protein in laryngeal cancer and normal tissues by IHC staining

**Figure 2:**
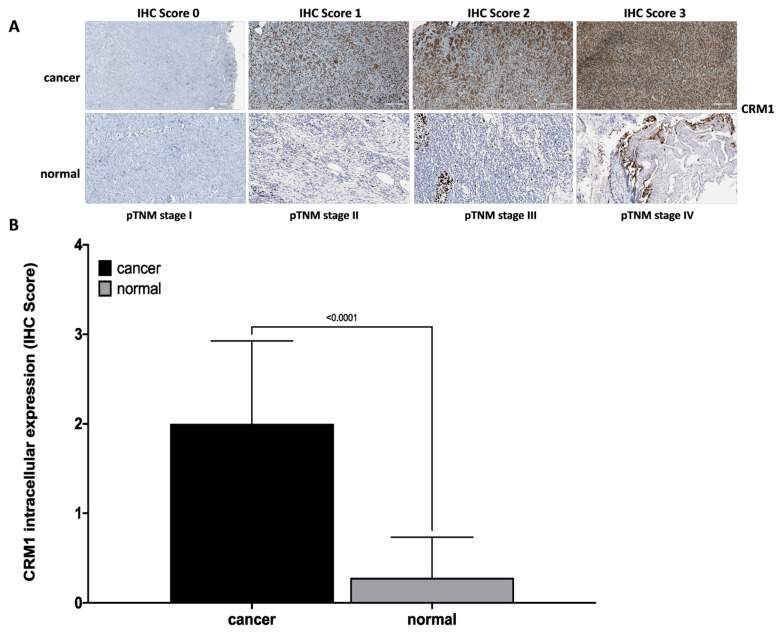
IHC data showing intracellular expression of CRM1 in matched tumor and normal tissue sections from laryngeal cancer patients. **(A)** Representative microscopic images of CRM1 staining scored by IHC method from laryngeal tumor and normal tissue sections at pTNM stage I, II, III, and VI (100× magnification, scale bar = 100 μm). CRM1 showed intense nuclear and cytoplasmic staining in tumor tissue and weak immunoreaction in normal laryngeal epithelium. In addition, as the tumor stage increased, an increase in the IHC score for CRM1 was observed. **(B)** IHC score data of CRM1 in matched tumor and normal tissues from laryngeal cancer patients. Intracellular expression of CRM1 was significantly higher in tumor tissues compared to normal tissues (p < 0.0001). IHC score data was obtained from at least three independent experiments. The intensity and amount of immunoreactivity in microscopic images obtained from cells stained with CRM1 antibody in ten randomly selected fields in each preparation were evaluated using Aperio ImageScope 12.4.3 software. Student’s t-test was used for statistical analysis. IHC score: 0 (no staining), +1 (weak staining), +2 (moderate staining), +3 (strong staining). Results are expressed as mean ± SD. CRM1: the chromosome region maintenance 1 protein, IHC: Immunohistochemical, SD: Standard error.

In IHC staining, CRM1 protein in laryngeal cancer tissue cells exhibited a strong cytoplasmic-nuclear staining. However, CRM1 showed weak and indistinct cytoplasmic-nuclear staining in paired normal laryngeal tissue (Figure 2). Increased cytoplasmic and nuclear staining of CRM1 was observed in advanced tumor tissue sections (Figure 2A). IHC scores were determined as “score 0” in 3 (7%), “score 1” in 9 (21%), “score 2” in 16 (37%), and “score 3” in 15 (35%) of tumor tissues of patients. Moreover, the score was “score 0” in 31 (%72) and “score 1” in 12 of the matched normal tissues. Consequently, it was observed that the intracellular expression of CRM1 was significantly increased in tumor tissues compared to matched normal tissues (Figure 2B).

### 3.4. Relationship between IHC staining and intracellular expression of CRM1 protein in laryngeal cancer tissues with clinicopathological features

If the IHC score is ≤1, intracellular expression of CRM1 was considered low, and if the score is ≥2, intracellular expression was considered high in laryngeal cancer tissues [24]. High CRM1 intracellular expression was statistically associated with increased tumor size (p < 0.001). At the same time, increased intracellular expression level of CRM1 was significantly associated with advanced tumor stage (p < 0.001), increased tumor invasion (p < 0.001), diffuse lymph node involvement (p < 0.001), distant metastasis (p = 0.026), vascular (p = 0.035) and perineural invasion (p = 0.008). However, no correlation was found between CRM1 intracellular expression level and other characteristics (p > 0.05).

A statistically significant increase in the intracellular expression of CRM1 was observed in stages IVa, IVb, IVc compared to pTNM stage I (p = 0.047, p = 0.013, p = 0.017, respectively), stage III, IVa, IVb, IVc compared to pTNM stage II (p = 0.009, p < 0.001, p = 0.005, p = 0.042, respectively), stage IVa compared to pTNM stage III (p = 0.003). The intracellular expression of CRM1 was statistically increased at T1, T2, T3, and T4 class compared to Tis class (p = 0.038, p < 0.001, p = 0.000, respectively), T3 and T4 class compared to T1 class (p = 0.006, p 0.008, respectively), T3 and T4 compared to T2 class (respectively, p = 0.011, p < 0.001), T4 compared to T3 class (p = 0.032). A significant increase in CRM1 intracellular expression was observed in classes N1, N2b, N3a (p = 0.021, p < 0.004, p < 0.001, respectively) compared to N0.

### 3.5. CRM1 protein expression in laryngeal cancer and normal tissues by Western-blot

**Figure 3:**
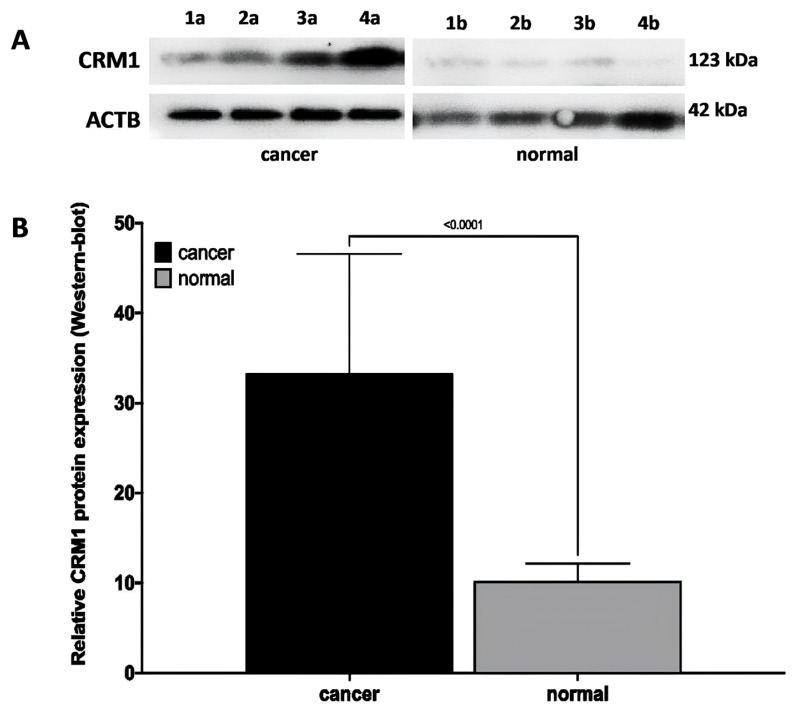
Western-blot data showing the CRM1 protein level in matched tumor and normal tissues of laryngeal cancer patients. **(A)** Representative CRM1 protein (123 kDa) band images of matched tumor (a) and normal (b) tissues of patients with pTNM stage I (1a–1b), II (2a–2b), III (3a–3b), and IV (4a–4b), respectively. CRM1 showed a dense immunoreactive band in tumor tissue and a weak immunoreactive band in normal laryngeal tissue. **(B)** Quantitative data of CRM1 protein bands in matched tumor and normal tissues from laryngeal cancer patients. The CRM1 protein level was significantly higher in tumor tissues compared to normal tissues (p < 0.0001). ACTB protein was used for normalization. Data was analyzed using ImageLab software. Densitometric analysis data are expressed as the mean ± SD of band intensities from three independent experiments. Student’s t-test was used for statistical analysis. ACTB: Beta-actin protein, CRM1: The chromosome region maintenance 1 protein, SD: Standard error

In western blot analysis, it was observed that CRM1 protein expression was increased in laryngeal cancer tissues, whereas protein expression was significantly decreased in matched normal laryngeal tissue (Figure 3). In advanced tumor tissues, an increase in the relative intensity of the immunoreactive band formed by the CRM1 protein was observed (Figure 3A). However, a statistically significant increase in the relative band density of the CRM1 protein by 3.23.02 ± 1.477 was observed in tumor tissues compared to matched normal tissues (Figure 3B).

### 3.6. Relationship between CRM1 protein expression and clinicopathological features in laryngeal cancer tissues by Western-blot

In tumor tissues of laryngeal cancer patients, if the densitometric data is ≤0.83, the CRM1 protein level is considered low, and if the data is >0.83, the protein level is considered high [34]. High CRM1 protein level was found to be statistically associated with distant metastasis, increased tumor size, and vascular and perineural invasion (p < 0.05). The increase in the protein level of CRM1 was also significantly associated with advanced tumor stage, increased tumor invasion, extensive lymph node involvement, distant metastasis, and advanced histological grade (p = 0.05) (Table 1). However, no correlation was found between CRM1 protein level and other features (p > 0.05).

A statistically significant increase was observed in CRM1 protein level in stages III, IVa, IVb compared to pTNM stage I (p = 0.030, p = 0.007, p < 0.001, respectively) and in stages IVa, IVb compared to pTNM stage II (p = 0.005, p < 0.001, respectively). A statistically significant increase was observed in CRM1 protein level at class T3 and T4 compared to Tis (p < 0.001, p < 0.001, respectively), in classes T3 and T4 compared to T1 (p < 0.001, p < 0.001, respectively), and in class T4 compared to T2 (p = 0.04). In addition, statistically significant increase in CRM1 protein level was observed in classes N1, N2a, N2b, N3a, N3b (p = 0.002, p = 0.001, p = 0.017, p < 0.001, respectively) compared to N0.

### 3.7. Best reference genes and their combinations for normalization in laryngeal cancer by NormFinder analysis

NormFinder analysis was employed to determine the reference gene combination with the lowest expression levels and the highest expression stability in laryngeal cancer. Stability value was calculated for each reference gene and gene combinations in the study group and ranked according to their stability (Table 2). As a result, *RPL29* (Stability value: 0.32) and *ALAS1* (Stability value: 0.32) genes were determined as the best reference genes and *ALAS1+RPL29* (Stability value: 0.23) as the best reference gene pair to be used in the study. The *ALAS1+RPL29* reference gene pair was used to normalize the qRT-PCR data to determine the mRNA level of *CRM1*.

### 3.8. *CRM1* mRNA expression in laryngeal cancer and normal tissues by qRT-PCR

**Figure 4:**
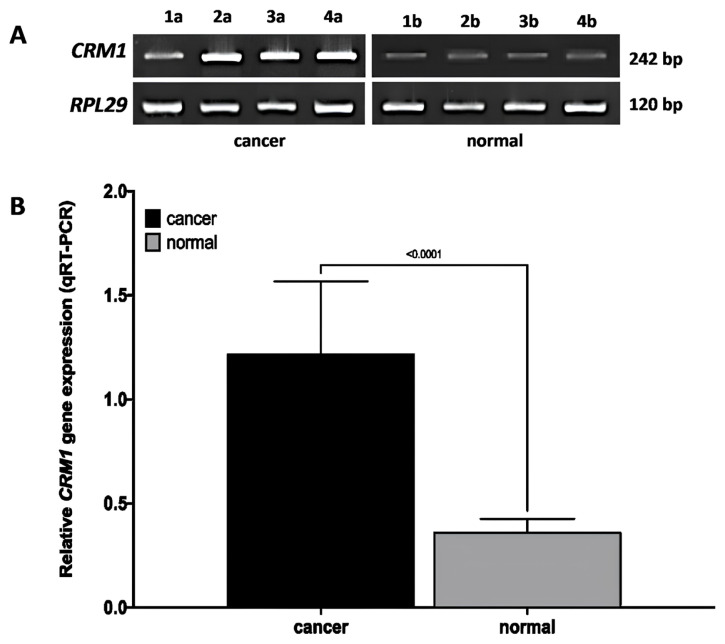
qRT-PCR data of *CRM1* gene mRNA level in matched tumor and normal tissues of laryngeal cancer patients. **(A)** Representative images of the qRT-PCR product (242 bp) of *CRM*1, obtained by agarose gel (5 μL/band) method, of matched tumor (a) and normal (b) tissues of patients with pTNM stage I (1a–1b), II (2a–2b), III (3a–3b), and IV (4a–4b), respectively. *RPL29* was used for normalization (RPL29 stability value: 0.32). *CRM1* showed a dense band in tumor tissue and a weak band in normal laryngeal tissue. **(B)** Quantitative data of *CRM1* gene mRNA level obtained by qRT-PCR method from matched tumor and normal tissues of laryngeal cancer patients. Combination of *ALAS1+RPL29* genes was used for normalization (*ALAS1+RPL29* stability value: 0.23). qRT-PCR data were obtained from three independent experiments and analyzed by the ^ΔΔ^CT method. Three wells were run for each cell group in independent experiments. Data were expressed as the relative change of ^ΔΔ^CT and the mean ± SD. Student’s t-test was used for statistical analysis. *ALAS1: 5’-Aminolevulinate Synthase 1*, *RPL29: Ribosomal Protein L29*, *CRM1: The chromosome region maintenance 1*, ^ΔΔ^CT: comparative CT method, SD: Standard error.

In qRT-PCR analysis, *CRM1* mRNA expression was increased in laryngeal cancer tissues, while mRNA expression was significantly decreased in matched normal laryngeal tissues (Figure 4). An increase in the band density formed by *CRM1* was observed in advanced tumor tissues (Figure 4A). Moreover, with quantitative data analysis, a significant increase at the rate of 4.00 ± 1.03 in the relative mRNA level of *CRM1* was observed in tumor tissues compared to matched normal tissues (Figure 4B).

### 3.9. Relationship of CRM1 gene mRNA expression with clinicopathological features in laryngeal cancer tissues by qRT-PCR

In tumor tissues of laryngeal cancer patients, if the quantitative data is ≤1.09, the *CRM1* mRNA level is considered low, and if the data is >1.09, the mRNA level is considered high [34]. High *CRM1* gene mRNA level was significantly associated with advanced tumor stage, increased tumor invasion, extensive lymph node involvement, and distant metastasis (p < 0.05) (Table 3). However, no correlation was found between *CRM1* gene mRNA expression level and other features (p > 0.05).

*CRM1* gene mRNA expression was statistically increased at pTNM stages IVa, IVb, IVc compared to stage I (p = 0.002, p = 0.001, p = 0.042, respectively), pTNM at stages IVa, IVb, IVc compared to stage II (p < 0.001, p < 0.001, p = 0.032, respectively). Also, statistically significant increase in *CRM1* gene mRNA expression was observed in class T3, T4 compared to Tis (p = 0.021, p = 0.026, respectively) and in class T3, T4 compared to T1 (p = 0.008, p = 0.013). A statistically significant increase in *CRM1* gene mRNA expression was observed in class N1, N2b compared to N0 (p = 0.008, p = 0.001, respectively).

### 3.10. Association between *CRM1* gene mRNA expression and prognosis of laryngeal cancer patients

**Figure 5:**
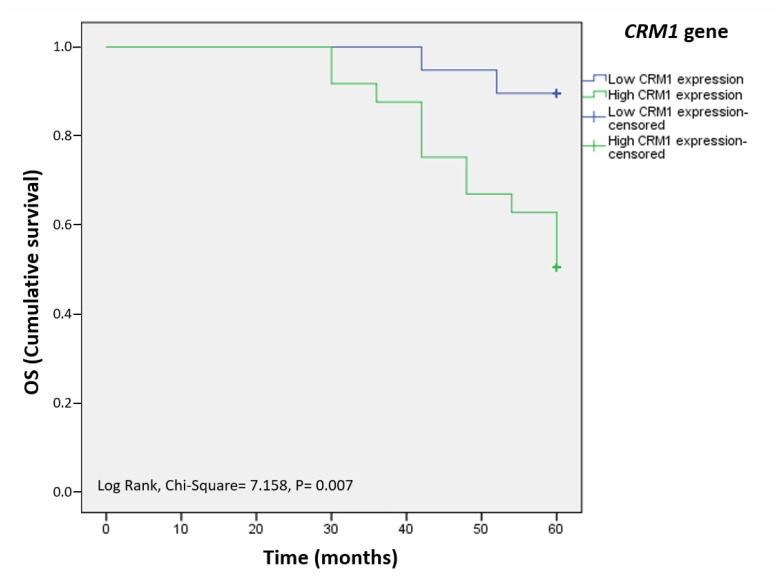
Overall survival curve for *CRM1* gene expression (low and high expression) in laryngeal cancer patients. Patients with higher *CRM1* gene mRNA (green line) had poorer survival compared to patients with lower *CRM1* gene mRNA (blue line) (p < 0.05). Kaplan–Meier and log-rank tests were used for statistical analysis and survival analysis. *CRM1: the chromosome region maintenance 1*, OS: Overall survival curve.

Death was observed in 14 of 43 patients (32.5%) with laryngeal cancer. Based on Kaplan–Meier survival analysis, the 5-year overall survival (OS) rate was 67.4%, with a median survival time of 55.4 months (range 52.8–58.1 months) [34]. The OS rate was worse in patients with a high *CRM1* mRNA level (>1.09) (50.0%) than in patients with a low *CRM1* gene mRNA level (≤1.0919) (89.5%) (Figure 5). However, the relationship between survival time and patients with high CRM1 intracellular (IHC Score ≥2) and protein (Western-blot >0.83) expression could not be evaluated statistically [24,34].

### 3.11. TCGA analysis

**Figure 6:**
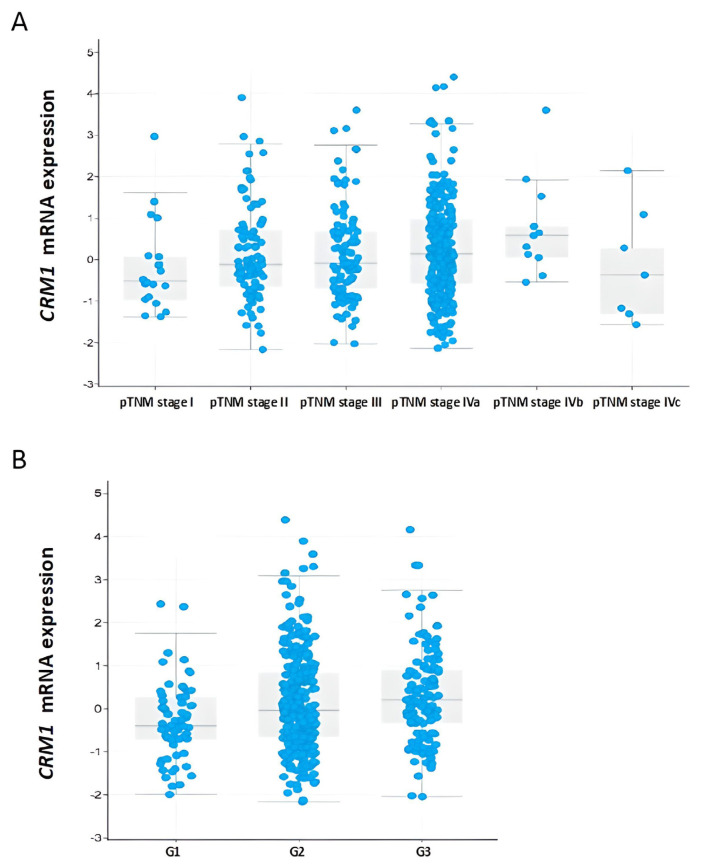
The relationship between *CRM1* mRNA level in head and neck cancer with clinical stage of tumor and histological grade (TCGA). **(A)** The relationship between *CRM1* mRNA level and pTNM clinical stage (p < 0.05). **(B)** The relationship between *CRM1* mRNA level and histological grade (G) (p < 0.05). The log2 mRNA expression levels (RNA Seq V2 RSEM) were obtained from TCGA data. Graph was plotted using cBioPortal to show the relationship between *CRM1* mRNA expression and clinical parameter (www.cbioportal.org). *CRM1*: *The chromosome region maintenance 1*, TCGA: The Cancer Genome Atlas.

Data analysis from cBioPortal (www.cbioportal.org) revealed a significant correlation between increase in *CRM1* gene mRNA expression and advanced tumor stage (pTNM stages IVa, IVb, IVc compared to stage I) and advanced histological grade (G2, G3 compared to G1) (p < 0.05) (Figure 6). Moreover, statistically significant increase in *CRM1* gene mRNA expression was observed in primary tumor stage (T3, T4, T4 compared to Tis and T1) (p < 0.05) and lymph node metastasis (N2, N2b, N2a compared to N0 and N1) (p < 0.05).

## 4. Discussion

In this study, gene expression in tumor and normal tissues was evaluated at the intracellular, protein, and mRNA levels to investigate the prognostic value of *CRM1* gene expression in laryngeal cancer. Consistent with the literature, in IHC staining, CRM1 protein showed a weak intracellular expression pattern in normal laryngeal tissue sections, while its expression in tumor tissue was quite strong [16,18,35]. In Western-blot analysis, high expression of CRM1 protein was observed in laryngeal cancer cells compared to normal tissue. Therewith, qRT-PCR analysis confirmed the IHC and western-blot data, showing increased expression of *CRM1* gene at the mRNA level in tumor tissue compared to normal tissue. In addition, increased *CRM1*, intracellular, protein, and mRNA expression in laryngeal tumor tissues was highly correlated with advanced histological grade, increased tumor size, advanced tumor stage, increased tumor invasion, diffuse lymph node involvement, distant metastasis, and vascular and perineural invasion. According to TCGA datasets, high *CRM1* gene expression in HNC including cases of squamous cell laryngeal origin was associated with advanced tumor stage and histological grade. Although its relationship with tumor stage and lymph node metastasis was not significant, it was a possible sign of poor prognosis. The results strengthen the idea that *CRM1* gene expression level may be an important risk factor for the clinical stage of the tumor, lymph node involvement, and distant metastasis in laryngeal cancer patients and may have a prognostic value in the clinic [16–18,35]. It can be used in the development of new treatment strategies for laryngeal cancer patients with increased *CRM1* expression level. Dysregulation of *CRM1* has been shown to be associated with increased expression in many types of cancer, particularly solid tumors [19]. As far as we know, this is the first study to report elevated *CRM1* expression as an independent prognostic marker for poor clinical course and overall survival in laryngeal cancer patients diagnosed with squamous cell carcinoma.

In the qRT-PCR method, the stability of reference genes is crucial for the accuracy of the relative quantitative analysis of target gene expression [28]. In our study, the expression stability of five reference genes *ACTB*, *ALAS1*, *GUSB*, *HMBS*, and *RPL29* were investigated to determine the *CRM1* gene expression profile in tumor tissues and normal tissues by NormFinder analysis [29]. We suggest that the combination of *ALAS1+RPL29* reference genes is best for normalization of target gene expression in laryngeal cancer and/or normal tissue samples [30,31].

Overexpression of *CRM1* is found in HNC [25,32], prostate cancer [13], breast cancer [14], cervical cancer [15], glioma [16], osteosarcoma [17], ovarian cancer [18], esophageal cancer [35], Kaposi’s sarcoma [36], pancreatic cancer [37], lung cancer [38], gastric cancer [39], renal cell carcinoma [39], hepatocellular carcinoma [41], acute myeloid/lymphoid leukemia [42], and chronic lymphoid leukemia [43], and this level of increase has been reported to be associated with metastasis, histological grade, increased tumor size, and lower overall survival. In head and neck squamous cell cancer cell lines, silencing of *CRM1* with small-interfering RNA (siRNA) and inhibiting protein function with Leptomycin B (LMB) inhibitor suppressed cell proliferation and cell migration, while promoting apoptosis [25,32]. Intracellular expression of CRM1 has been reported to be associated with advanced tumor stage and poor survival in ovarian cancer with IHC. It has also been reported that inhibition of CRM1 with LMB in in vitro cell lines reduces the expression of Cyclooxygenase (COX)-2, suppresses cell proliferation, and promotes apoptosis [18]. Along with existing studies, increased *CRM1* expression has been reported in tumor tissue compared to the normal tissue of patients with gastric cancer and osteosarcoma, and high expression level has been reported to be associated with clinicopathological parameters and overall survival [17,39]. In a study of breast cancer, Western-blot data with IHC showed increased expression of CRM1 in breast cancer tissues compared to normal tissues, and this increase was associated with tumor size, advanced tumor stage, tumor invasion, extensive lymph node involvement, distant metastasis, and poor survival [44].

An in vitro study demonstrated that a Selinexor-derived synthetic inhibitor suppressed dose-dependent cell proliferation, promoted apoptotic pathways in hepatocellular carcinoma cells with suppressing CRM1 protein function [45]. It has been reported that Nuclear transcription factor Y (NFY)/CREB-binding protein (CBP), Sp1 transcription factor (SP1), and tumor protein p53 (P53) transcription factors interact with the promoter of the *CRM1* gene and together play an important role in the transformation of cancer cells [46]. In previous studies, it has been reported that the specific inhibition of CRM1 increases the nuclear uptake and expression of major tumor suppressor proteins such as RB, P53, P21, P33, FOXO, P27, and decreases the expression of oncogenic proteins such as MYC, MET, and EGFR [9,10,19]. CRM1 is the solo nuclear transporter of many proteins with prognostic value in laryngeal cancer, such as P53, P16, P63, Rb, PTEN, Nuclear factor kappa B (NFκB), EGFR, Cyclin D1, Survivin, and BCL-2 [9,19,46,47]. In addition, in vitro and in vivo studies and bioinformatic analyses have shown that *CRM1* promotes cancer in the cell by suppressing the immune response in the microenvironment through various biological pathways [10,48]. Our study results showed that increased expression of *CRM1* in laryngeal cancer was associated with advanced histological grade. Our data confirmed that increased *CRM1* expression is an important risk factor for tumor clinical stage, tumor size, primary tumor invasion, lymph node metastasis, distant metastasis, and vascular and perineural involvement in laryngeal cancer patients. In conclusion, increased *CRM1* expression level in laryngeal cancer may be an indicator of poor prognosis.

By inhibiting CRM1 function, enabling various tumor suppressor proteins to accumulate in the nucleus, and activating apoptotic pathways may enable the development of new molecule-targeted therapy strategies [49]. Recently, several synthetic CRM1-inhibitors have been developed, such as the new generation PKF050-638, CBS9106, and Selective Nuclear Export inhibitors (SINEs) including KPT-185, KPT-276, Verdinexor (KPT-335), Selinexor (KPT-330), Eltanexor (KPT-8602), and Felezonexor (SL-801) which are less toxic, semireversible inhibitors instead of the first inhibitor LMB [19,50]. A number of SINE compounds, showing efficacy in solid tumors [44,50] and hematopoietic malignancies [41,52,53], have been extensively tested in preclinical settings. Promising advances have been reported in the clinical use of SINE derivatives KPT-330, KPT-8602, and SL-80, and especially the use of KPT-330 as a CRM1 inhibitor in the treatment of multiple myeloma has been approved by the Food and Drug Administration (FDA) [19]. Dysregulation of CRM1-dependent nuclear-cytoplasmic transport, which is part of normal cell function, in carcinogenesis offers a unique therapeutic opportunity and enables inhibitors to selectively target cancer cells.

## 5. Conclusion

In conclusion, this study revealed that *CRM1* is a predictive biomarker for poor overall survival in laryngeal cancer patients, and that the evaluation of the clinical pTNM staging system of the tumor and *CRM1* expression together may provide additional prognostic information. Moreover, inhibition of *CRM1* expression may be a new therapeutic strategy for laryngeal cancer. Furthermore, prospective large-scale studies are needed to explore the true prognostic role of elevated *CRM1* expression in laryngeal squamous cell carcinoma due to the limited sample size.

## Figures and Tables

**Table 1:** Correlations between CRM1 protein expression level and clinicopathological variables in laryngeal cancer.

Clinicopathological variables		Total n (%)	Low expression n (%)	High expression (%)	χ^2^	p	Expression level mean ± SD	p
**Sex**	Male	40 (93)	20 (50.0)	20 (50.0)	0.31	0.999	0.76 ± 0.36	0.70
	Female	3 (7)	2 (66.7)	1 (33.3)	**-**	**-**	0.68 ± 0.50	**-**
**Age at surgery (years)**	<60	17 (39.5)	12 (70.6)	5 (29.4)	4.25	0.062	0.67 ± 0.38	0.27
	≥60	26 (60.5)	10 (38.5)	16 (61.5)	**-**	**-**	0.81 ± 0.35	**-**
**Tumor location**	Glottic	28 (65.2)	17 (60.7)	11 (39.3)	3.08	0.248	0.71 ± 0.39	0.74
	Subglottic	4 (9.3)	1 (25.0)	3 (75.0)	-	-	0.81 ± 0.33	-
	Supraglottic	11 (25.5)	4 (36.4)	7 (63.6)	-	-	0.85 ± 0.32	-
**pTNM stage**	I	4 (9.3)	4 (100)	0 (0.0)	NA	NA	0.33 ± 0.02	
	II	13 (30.2)	12 (92.3)	1 (7.7)	-	-	0.47 ± 0.25	-
	III	10 (23.2)	5 (50.0)	5 (50.0)	-	-	0.82 ± 0.36	-
	IVa	6 (14)	0 (0.0)	0 (0.0)	-	-	1.09 ± 0.23	-
	IVb	6 (14)	1 (16.7)	5 (83.3)	-	-	1.00 ± 0.09	-
	IVc	4 (9.3)	0 (0.0)	4 (100)	-	-	1.06 ± 0.21	-
**T classification**	Tis	3 (7)	3 (100)	0 (0.0)	NA	NA	0.36 ± 0.06	*0.001*
	T1	5 (11.6)	5 (100)	0 (0.0)	-	-	0.37 ± 0.12	-
	T2	14 (32.5)	10 (71.4)	4 (28.6)	-	-	0.64 ± 0.39	-
	T3	14 (32.5)	4 (28.6)	10 (71.4)	-	-	0.95 ± 0.29	-
	T4	7 (16.4)	0 (0.0)	7 (100)	-	-	1.03 ± 0.14	-
**Lymph node metastasis**	N0	19 (44.2)	19 (100)	0 (0.0)	NA	NA	0.44 ± 0.21	*<0.001*
	N1	10 (23.2)	3 (30.0)	7 (70.0)	-	-	0.90 ± 0.23	*-*
	N2a	1 (2.3)	0 (0.0)	1 (100)	-	-	1.34 ± 0.00	*-*
	N2b	6 (14)	0 (0.0)	6 (100)	-	-	1.18 ± 0.21	*-*
	N3a	3 (7)	0 (0.0)	3 (100)	-	-	0.98 ± 0.11	*-*
	N3b	4 (9.3)	0 (0.0)	4 (100)	-	-	1.02 ± 0.10	*-*
**Distant metastasis**	M0	31 (72)	22 (71.0)	9 (29.0)	17.44	*<0.001*	0.61 ± 0.30	*<0.001*
	M1	12 (28)	0 (0.0)	12 (100)	-	-	1.14 ± 0.21	*-*
**Histologic grade**	G1	17 (39.5)	12 (70.6)	5 (29.4)	4.62	0.111	0.59 ± 0.31	*0.04*
	G2	19 (44.2)	8 (42.1)	11 (57.9)	-	-	0.81 ± 0.33	*-*
	G3	7 (16.3)	2 (28.6)	5 (71.4)	-	-	1.01 ± 0.42	*-*
**Size of primary tumor (cm** **^3^** **)**	≤5	24 (55.8)	21 (87.5)	3 (12.5)	28.70	*<0.001*	0.53 ± 0.30	*<0.001*
	>5	19 (44.2)	1 (5.3)	18 (94.7)	-	-	1.04 ± 0.21	*-*
**Vessel invasion**	Positive	9 (21)	0 (0.0)	9 (100)	11.92	*0.001*	1.14 ± 0.23	*0.001*
	Negative	34 (79)	22 (64.7)	12 (35.3)	-	-	0.65 ± 0.32	*-*
**Perineural invasion**	Positive	8 (18.6)	1 (12.5)	7 (87.5)	5.19	*0.046*	0.94 ± 0.20	0.13
	Negative	(81.4)	21 (60.0)	14 (40.0)	-	*-*	0.71 ± 0.38	-

* CRM1 protein expression level was considered low if the Western-blot was ≤0.8282 and high if it was >0.8282.

CRM1: the chromosome region maintenance 1 protein, SD: Standard error, NA: not analyzed.

**Table 2:** Stability values of reference genes and in-group reference gene combinations with NormFinder.

No	Reference gene	Gene stability value	Reference gene 1 + gene 2	Gene combinations stability value
**1**	*RPL29*	0.32^*^	*ALAS +1RPL29*	0.23^*^
**2**	*ALAS1*	0.32^*^	*HMBS + RPL29*	0.24
**3**	*HMBS*	0.33	*ALAS1+HMBS*	0.25
**4**	*ACTB*	0.41	*ACTB + RPL29*	0.29
**5**	*GUSB*	0.46	*ACTB + ALAS1*	0.31
**6**			*ACTB + HMBS*	0.32

RPL29: Ribosomal Protein L29, ALAS1:5′-Aminolevulinate Synthase 1, HMBS: Hydroxymethylbilane Synthase, ACTB: Actin Beta, GUSB: Glucuronidase Beta.

**Table 3:** Correlations between *CRM1* gene mRNA expression level and clinicopathological variables in laryngeal cancer.

Clinicopathological variables		Totaln (%)	Low expressionn (%)	High expression (%)	χ^2^	p	Expression levelmean ± SD	p
**Sex**	Male	40 (93)	16 (40.0)	24 (60.0)	0.82	0.56	1.23 ± 0.35	0.50
	Female	3 (7)	2 (66.7)	1 (33.3)	**-**	**-**	1.09 ± 0.24	**-**
**Age at surgery (years)**	<60	17 (39.5)	9 (52.9)	8 (41.7)	1.42	0.34	1.19 ± 0.37	0.61
	≥60	26 (60.5)	9 (34.6)	17 (65.4)	**-**	**-**	1.24 ± 0.33	**-**
**Tumor location**	Glottic	28 (65.2)	14 (50.0)	14 (50.0)	2.19	0.40	1.21 ± 0.37	0.45
	Subglottic	4 (9.3)	1 (25.0)	3 (75.0)	**-**	**-**	1.45 ± 0.42	**-**
	Supraglottic	11 (25.5)	3 (27.3)	8 (72.7)	**-**	**-**	1.15 ± 0.23	**-**
**pTNM stage**	I	4 (9.3)	4 (100)	0 (0.0)	NA	NA	0.87 ± 0.02	*<0.001*
	II	13 (30.2)	11 (84.6)	2 (15.4)	**-**	**-**	0.96 ± 0.24	**-**
	III	10 (23.2)	3 (30.0)	7 (70.0)	**-**	**-**	1.24 ± 0.31	**-**
	Ia	6 (14)	0 (0.0)	6 (100)	**-**	**-**	1.53 ± 0.22	**-**
	Ib	6 (14)	0 (0.0)	6 (100)	**-**	**-**	1.57 ± 0.26	**-**
	Ic	4 (9.3)	0 (0.0)	4 (100)	**-**	**-**	1.40 ± 0.16	**-**
**T classification**	Tis	3 (7)	3 (100)	0 (0.0)	NA	NA	0.83 ± 0.07	*<0.001*
	T1	5 (11.6)	5 (100)	0 (0.0)	**-**	**-**	0.88 ± 0.10	**-**
	T2	14 (32.5)	8 (57.1)	6 (42.9)	**-**	**-**	1.12 ± 0.34	**-**
	T3	14 (32.5)	2 (14.3)	12 (85.7)	**-**	**-**	1.41 ± 0.29	**-**
	T4	7 (16.4)	0 (0.0)	7 (100)	**-**	**-**	1.45 ± 0.25	**-**
**Lymph node metastasis**	N0	19 (44.2)	17 (89.5)	2 (10.5)	NA	NA	0.93 ± 0.18	*<0.001*
	N1	10 (23.2)	1 (10.0)	9 (90.0)	**-**	**-**	1.43 ± 0.30	**-**
	N2a	1 (2.3)	0 (0.0)	1 (100)	**-**	**-**	1.44 ± 0.00	**-**
	N2b	6 (14)	0 (0.0)	6 (100)	**-**	**-**	1.47 ± 0.21	**-**
	N3a	3 (7)	0 (0.0)	3 (100)	**-**	**-**	1.56 ± 0.26	**-**
	N3b	4 (9.3)	0 (0.0)	4 (100)	**-**	**-**	1.47 ± 0.27	**-**
**Distant metastasis**	M0	31 (72)	18 (58.1)	13 (41.9)	11.99	*<0.001*	1.11 ± 0.32	*<0.001*
	M1	12 (28)	0 (0.0)	12 (100)	**-**	**-**	1.515 ± 0.203	**-**
**Histologic grade**	G1	17 (39.5)	11 (64.7)	6 (35.3)	6.66	*0.05*	1.07 ± 0.33	*0.03*
	G2	19 (44.2)	6 (31.6)	13 (68.4)	**-**	**-**	1.27 ± 0.31	**-**
	G3	7 (16.3)	1 (14.3)	6 (85.7)	**-**	**-**	1.45 ± 0.37	**-**
**Size of primary tumor (cm** **^3^** **)**	≤ 5	24 (55.8)	17 (70.8)	7 (29.2)	18.73	*<0.001*	0.97 ± 0.26	*<0.001*
	> 5	19 (44.2)	1 (5.3)	18 (94.7)	**-**	**-**	1.42 ± 0.25	**-**
**Vessel invasion**	Positie	9 (21)	0 (0.0)	9 (100)	8.19	*0.01*	1.42 ± 0.19	0.06
	Negatie	34 (79)	18 (52.9)	16 (47.1)	-	*-*	1.17 ± 0.36	-
**Perineural invasion**	Positive	8 (18.6)	1 (12.5)	7 (87.5)	3.48	0.11	1.42 ± 0.27	0.06
	Negative	81.4)	17 (48.6)	18 (51.4)	-	*-*	1.17 ± 0.35	-

* *CRM1* gene mRNA expression level was considered low if the qRT-PCR was ≤1.0919 and high if it was >1.0919.

*CRM1: the chromosoe region maintenance 1 protein*, qRT-PCR: Quantitative real–time RT–PCR, SD: Standard error, NA: not analyzed.
